# Association Between Social Media Use and Burnout Among Primary Health Care Workers During the COVID-19 Pandemic in China: Nationwide Cross-Sectional Survey

**DOI:** 10.2196/70398

**Published:** 2025-07-31

**Authors:** Jie Gu, Ping Zhu, Yijing Chu, Yuge Yan, Yuqi Yang, Jing Guo, Biao Xi, Shanzhu Zhu, Hong Liang, Jiewen Xiao, Jiaoling Huang

**Affiliations:** 1 Department of General Practition, Zhongshan Hospital Fudan University, Shanghai, China; 2 International Medical Center, Zhongshan Hospital Fudan University, Shanghai, China; 3 School of Public Health, Shanghai Jiao Tong University School of Medicine, No. 270 South Chongqing Road, Shanghai, 200025, China, 86 13916645705; 4 Shanghai Institute of Infectious Disease and Biosecurity, Fudan University, Shanghai, China; 5 School of Social Development and Public Policy, Fudan University, Shanghai, China; 6 Hebei Medical University, Shijiazhuang, China; 7 Shanghai Baoshan District Wusong Central Hospital, Shanghai, China

**Keywords:** burnout, China, primary health care workers, social media, urban-rural difference

## Abstract

**Background:**

The COVID-19 pandemic has intensified health care worker burnout and increased their engagement with social media. However, it remains unclear whether social media is beneficial in mitigating burnout among primary health care workers (PHWs).

**Objective:**

This study aims to report the prevalence of burnout among Chinese PHWs and investigate the relationship between social media use, specifically WeChat Moments usage, and burnout, with a focus on urban-rural differences.

**Methods:**

Our nationwide cross-sectional survey was conducted between May and October 2022 and included 3769 PHWs. Burnout was assessed using the Maslach Burnout Inventory-Human Services Survey, and WeChat Moments usage was self-evaluated. Weighted data were used to report the prevalence of burnout nationwide and in urban and rural areas. Multivariate logistic regression and subgroup analyses were used to examine the association between burnout and WeChat Moments usage frequency, highlighting differences between urban and rural PHWs.

**Results:**

Urban PHWs reported a higher prevalence of burnout compared with their rural counterparts (212/1200, 17.6% vs 351/2569, 13.7%; *P*=.004). Overall, the frequency of social media use was negatively associated with the prevalence of burnout. With the inclusion of covariates, those who sometimes used WeChat Moments experienced a statistically significant protective effect compared with those who never used WeChat Moments in the fully adjusted model (odds ratio 0.570, 95% CI 0.348‐0.933; *P*=.03). The association between social media use and burnout was more statistically significant in urban settings than in rural settings (urban: all odds ratios and 95% CIs <1, and all *P*<.05; rural: all *P*>.05).

**Conclusions:**

Urban and rural differences in burnout prevalence were observed among PHWs, with urban practitioners experiencing significantly higher prevalence. This study also found that increased social media use was associated with a lower likelihood of experiencing burnout, but the association did not always exist. In terms of overall burnout, the relationship between social media use and burnout was statistically significant only in urban areas. Our findings underscore the urgent need for policy makers and health care institutions to prioritize interventions addressing burnout among PHWs, particularly in underresourced and high-stress settings. Local governments could pilot platforms with moderation, allowing PHWs to showcase their work progress to the communities they serve, thereby strengthening trust and reducing emotional exhaustion. Our research also suggests that social media interventions may be particularly effective in urban settings. These findings offer actionable insights for other low- and middle-income countries navigating similar challenges. International bodies (eg, the World Health Organization) should develop digital health guidelines specific to low- and middle-income countries to help policy makers balance the benefits and risks of social media.

## Introduction

Burnout is a common problem that has garnered significant attention from researchers over the past decade. The number of articles about burnout-related topics has increased exponentially [1]. Burnout is a syndrome that encompasses emotional exhaustion (EE), depersonalization (DP), and reduced personal accomplishment (PA) [2]. Among health care workers, burnout can directly affect their health status, the quality of health care, patient satisfaction, and even patient safety [3-5]. This phenomenon is particularly pronounced among primary health care workers (PHWs) in China. The term “PHWs” refers to clinically qualified staff working in primary health care (PHC) facilities. During the COVID-19 pandemic, more than 50% of PHWs reported experiencing burnout [6-9]. Despite the prevalence of burnout among PHWs, they often receive insufficient attention [10].

During the COVID-19 pandemic, health care workers exhibited heightened engagement with social media platforms, a trend attributed to the escalation of social distancing measures and a concurrent reduction in traditional social interactions [11,12]. Social media provides a platform for sharing information and connecting with others, which can help reduce stress among health care workers [12,13], support their personal development [14], and facilitate knowledge exchange [15]; however, it can also disseminate false or misleading content, which may increase anxiety [16]. Additionally, the overwhelming amount of information on social media [15], privacy concerns [17], and the blurring of boundaries between work and personal life [18,19] can all contribute to negative outcomes. Importantly, existing studies have mainly examined Western social media platforms (eg, Facebook [20,21] and Instagram [22,23]), and there is a lack of understanding of China’s dominant social media platform, WeChat, which has 1.3 billion users worldwide [24].

WeChat integrates various functionalities, including instant messaging, social sharing (WeChat Moments), WeChat Pay, official accounts, and mini-programs. The Health Commission of Hangzhou, China, previously developed a WeChat-based mini-program that enabled online access to family doctors, notably featuring direct WeChat contact addition functionality [25]. Meanwhile, Anhui Provincial Health Commission reported that family doctors adopted a work approach using “family doctor WeChat groups” for efficient health management [26]. In this context, we focus on the unique social sharing feature “WeChat Moments” as a measure of social media engagement. WeChat Moments is akin to early versions of Facebook or Instagram and is characterized by its emphasis on acquaintance-based social interactions. WeChat Moments allows users to share and repost text, photos, and videos with mutual friends, who can then like and comment on these posts [27], and this may enhance social support and social connection among users.

WeChat’s “Moments” feature (a closed-network sharing space for verified contacts) has unique characteristics that may differentially influence burnout. Unlike Western social media platforms that allow open networking, WeChat Moments prioritizes interactions within established social circles, potentially amplifying social comparison pressures among PHWs [28]. Preliminary findings are contradictory. A cross-sectional study of rural doctors in China reported that those who found WeChat difficult to use were more likely to experience burnout (odds ratio 1.436, 95% CI 1.229‐1.679; *P*<.001) [6]. However, studies on closed Facebook groups with features similar to WeChat Moments indicated that social media use may also help alleviate burnout [20,21]. While it can partially reduce feelings of professional isolation, the overall impact on mitigating burnout remains uncertain [20,21]. The previous cross-sectional study on the association between WeChat use difficulties and burnout among physicians in rural China during COVID-19 also suggested that we need to pay attention to the differences in the association between social media use and burnout in urban and rural China [6].

Job burnout manifests as a critical occupational hazard among PHWs, particularly within the COVID-19 high-pressure health care environment. Although prior studies have established the epidemiological prevalence of burnout syndrome, two underexamined determinants need to be discussed: (1) the frequency of WeChat Moments engagement as a risk factor of burnout, and (2) disparities between urban and rural settings. To address these gaps, this study aims to explore the relationship between social media engagement and burnout, with a specific focus on WeChat Moments and the urban and rural contexts. Specifically, this study has the following three research objectives: (1) to report the prevalence of burnout at the national level in China, (2) to investigate the relationship between the frequency of WeChat Moments usage and multidimensional burnout among PHWs, and (3) to identify urban-rural differences in the relationship.

## Methods

### Study Design and Participant Recruitment

Our study was conducted between May and October 2022 and employed a nationwide cross-sectional online survey strategy that aimed to capture the association between burnout and social media use among PHWs across China’s diverse provinces, excluding Hong Kong, Macao, and Taiwan. The second half of 2022 marked the tail end of the COVID-19 pandemic, with the pandemic control measures in place for 3 years beginning to be relaxed, offering a unique window into the impact of prolonged public health measures on the well-being of PHWs. The study included personnel with clinical qualifications from community health centers (CHCs) and their affiliated stations, township hospitals, or village clinics. Participants were required to possess a smartphone and have the ability to use questionnaire filling functions. The study excluded nonclinical personnel directly involved in outpatient or inpatient diagnosis and treatment, public health services, or nursing work (eg, administrative, logistical, and financial work).

We first used a multistage sampling approach to ensure a representative sample of PHC facilities across urban and rural settings. Initially, we delineated the provinces based on China’s administrative divisions. Within each province, we identified the provincial capital cities. A sampling frame of nonprovincial capital cities was created, and a random sample of nonprovincial capital cities was selected using a random number table. Urban PHC systems typically consist of CHCs and their affiliated stations, while rural systems comprise township hospitals and village clinics. For provincial capital cities, we established a sampling frame of all CHCs and their affiliated stations within the city, from which a CHC and its affiliated stations were randomly selected using a random number table. For nonprovincial capital cities, we similarly created a sampling frame of all township hospitals and their affiliated village clinics within the city, from which a township hospital and its affiliated village clinics were randomly selected using a random number table. Given the ongoing urbanization and the transformation of some township hospitals into CHCs, we adapted our sampling approach to include CHCs in such cases. In the 4 municipalities of Beijing, Shanghai, Chongqing, and Tianjin, we randomly selected 2 CHCs each, one from an urban area and another from a suburban area. If there were cases of noncooperation from the sampled health care organizations, a second round of sampling was conducted. Finally, we selected 62 PHCs in total, encompassing 44 CHCs and 18 township hospitals.

We then adopted the method of cluster sampling. Through the directors of the sampled PHCs, the participants received an online questionnaire that had been set up on the Questionnaire Star website [29]. All PHWs working at the PHCs were invited. Before filling out the questionnaire, we obtained informed consent from each participant through online selection options, ensuring the confidentiality and anonymity of the data. Ultimately, 3769 PHWs (93.7%) participated in this survey. According to post hoc power calculations, the statistical power exceeded the conventional threshold of 0.80 at *α*=.05 based on the observed effect size (Cohen *w*=0.079) and total sample size (N=3769). This study followed the STROBE (Strengthening the Reporting of Observational Studies in Epidemiology) reporting guidelines (Checklist 1).

### Measures and Variables

#### Job Burnout

The Chinese version of the Maslach Burnout Inventory-Human Services Survey was used to measure burnout (Cronbach α coefficients of 0.89 for EE, 0.79 for DP, and 0.87 for PA) [30].

This questionnaire consists of 22 questions and 3 subscales (EE, DP, and PA), and a 7-point Likert scale is used for assessment. The scores of individual questions are added, and the findings are used to evaluate overall burnout and the situation of various dimensions of burnout. We used the commonly adopted cutoff values for this questionnaire to assess the following aspects: overall burnout (EE score ≥27, DP score ≥10, and PA score ≤33), high EE (EE score ≥27), high DP (DP score ≥10), and low PA (PA score ≤33) [31].

#### Social Media WeChat Use

Participants were required to describe the frequency of WeChat Moments posting on WeChat by choosing “Never,” “Seldom,” “Occasionally,” “Sometimes,” or “Usually,” and the survey assessed general WeChat Moments usage frequency rather than restricting the evaluation to a specific period (eg, COVID-19 peak or during the past month).

#### Covariates

Covariates were chosen based on the literature [2,32], and the following 4 dimensions were considered: sociodemographic factors, individual health factors, occupational status factors, and COVID-19 pandemic factors. The *sociodemographic factors* dimension included gender, age, education level, marriage status, and family relations. These variables are commonly identified as potential confounders or moderators in studies of mental health and burnout, allowing us to control for background characteristics and explore their modifying effects. The *individual health factors* dimension included smoking, drinking, number of diseases, disability, and self-rated health. These factors reflect baseline physical and mental health, which can influence susceptibility to burnout. The *occupational status factors* dimension included occupation category, professional title, length of career in primary care, and monthly income. These are direct indicators of job demand, resources, and perceived work-life balance, all of which are well-established determinants of burnout in health care settings. The *COVID-19 pandemic factors* dimension was described in terms of 4 aspects, including frontline health care workers, satisfaction with institutional provision of personal protective equipment, self-rated work intensity during COVID-19, and the Minnesota Satisfaction Questionnaire-Short Form (MSQ-SF). These variables were specifically chosen to capture the unique stressors and protective factors experienced by health care workers during the pandemic, directly addressing our research question regarding the pandemic’s impact. Regarding the satisfaction with institutional provision of personal protective equipment, the following question was considered: “During the COVID-19 pandemic, how satisfied were you with the personal protective measures (eg, masks, gloves, isolation gowns, and disinfectants) provided by your workplace? (1‐5 scale, where 1 indicates ‘Very dissatisfied’).” The MSQ-SF was used to assess job satisfaction. It consists of 20 items measuring satisfaction on a 5-point Likert scale (ranging from 1 [“Very dissatisfied”] to 5 [“Very satisfied”]), with higher scores indicating higher job satisfaction.

### Statistical Analysis

We performed weighted analysis based on the data of PHWs from the Statistical Bulletin on the Development of China’s Health and Wellness Industry in 2022 [31]. We used the *χ*^2^ test to assess the association between the category of social media use and burnout. Multivariate logistic regression models were specified to assess the association of burnout with WeChat Moments usage after controlling for covariates. The first model was adjusted for sociodemographic factors (age, gender, marital status, education, living arrangement, and family relations). The second model was adjusted for sociodemographic factors and individual health factors. The third model was adjusted for sociodemographic factors, individual health factors, and occupational status factors. The fourth model was adjusted for sociodemographic factors, individual health factors, occupational status factors, and COVID-19 pandemic factors. Finally, the multivariate logistic regression results were analyzed in each subgroup to identify the difference in WeChat Moments usage between rural and urban areas. Bonferroni correction was used, and the corrected significance level was .025. The findings have been presented in a forest plot. Statistical analysis was conducted using IBM SPSS statistical software, version 22 (IBM Corp).

### Ethical Considerations

This study was approved by the Institutional Research Board of Zhongshan Hospital, Fudan University (approval number: B2021-605). Written informed consent was obtained from all participants prior to data collection. To protect privacy and confidentiality, all participant data were deidentified by removing direct identifiers (eg, names and contact information) before analysis. No financial compensation was provided, as the study required minimal time involvement and posed no physical risks. Additionally, the manuscript and supplementary materials contain no identifiable personal information, including images, videos, or location data.

## Results

### Characteristics and Prevalence of Burnout

Among the 3769 participants in the weighted population (rural: 2569, urban: 1200), 562 (14.9%) had overall burnout, 1014 (26.9%) had EE, 1013 (26.9%) had DP, and 2353 (62.4%) had low PA (Table 1). The characteristics of the unweighted population (rural: 989, urban: 2780) are provided in Multimedia Appendix 1. In this population, marital status, living arrangements, family relationships, disability status, the status of being a frontline health worker, and the MSQ-SF score were not significantly different between rural and urban areas (all *P*>.05). In the weighted population, 52.8% (1356/2569) of rural PHWs and 48.5% (583/1200) of urban PHWs occasionally used WeChat Moments.

**Table 1.** Characteristics of the participants in the weighted population (N=3769).

Characteristic	Weighted population			*P* value
	Total (N=3769)	Rural (n=2569)	Urban (n=1200)	
Gender, n (%)				<.001
Male	1001 (26.6)	787 (30.6)	214 (17.8)	
Female	2768 (73.4)	1782 (69.4)	987 (82.2)	
Age (years), n (%)				.005
≤30	1023 (27.1)	722 (28.1)	301 (25.0)	
31‐40	1421 (37.7)	917 (35.7)	504 (42.0)	
41‐50	908 (24.1)	634 (24.7)	275 (22.9)	
>50	417 (11.1)	296 (11.5)	121 (10.1)	
Marital status, n (%)				.46
Single	622 (16.5)	410 (16.0)	212 (17.7)	
Married	2948 (78.2)	2021 (78.7)	928 (77.3)	
Divorced/widowed	199 (5.3)	138 (5.4)	61 (5.1)	
Educational status, n (%)				<.001
High school or below	665 (17.6)	587 (22.9)	78 (6.5)	
Junior college	1121 (29.7)	758 (29.5)	363 (30.2)	
Undergraduate or above	1983 (52.6)	1223 (47.6)	760 (63.3)	
Disability	771 (20.5)	493 (19.2)	278 (23.1)	.01
Occupation category, n (%)				<.001
GP^a^	1412 (37.5)	1086 (42.3)	326 (27.2)	
Nurse	1091 (28.9)	644 (25.1)	446 (37.2)	
Public health physician	243 (6.5)	161 (6.3)	82 (6.9)	
Managerial staff	195 (5.2)	119 (4.7)	76 (6.3)	
Support staff	828 (22.0)	558 (21.7)	269 (22.4)	
MSQ-SF^b^ score, mean (SD)	70 (13)	70 (13)	70 (13)	.32
WeChat Moments usage, n (%)				.005
Never	136 (3.6)	99 (3.8)	38 (3.1)	
Seldom	466 (12.4)	291 (11.3)	175 (14.6)	
Occasionally	1938 (51.4)	1356 (52.8)	583 (48.5)	
Sometimes	828 (22.0)	538 (20.9)	290 (24.2)	
Usually	401 (10.6)	286 (11.1)	115 (9.6)	
Burnout, n (%)				
Overall	562 (14.9)	351 (13.7)	212 (17.6)	.004
Emotional exhaustion	1014 (26.9)	636 (24.8)	378 (31.5)	<.001
Depersonalization	1013 (26.9)	667 (26.0)	345 (28.8)	.09
Low personal accomplishment	2353 (62.4)	1595 (62.1)	759 (63.2)	.53

a GP: general practitioner.

b MSQ-SF: Minnesota Satisfaction Questionnaire-Short Form.

### Association Between WeChat Moments Usage and Burnout

We found that WeChat Moments usage was associated with overall burnout (*χ*^2^_4_=23.790; *P*<.001), EE (*χ*^2^_4_=23.624; *P*<.001), DP (*χ*^2^_4_=25.727; *P*<.001), and low PA (*χ*^2^_4_=13.133; *P*=.01) (Table 2). WeChat Moments usage was negatively associated with burnout. Compared with participants who used WeChat Moments less frequently (never, seldom, and occasionally), those who used it more frequently (sometimes and usually) had a lower incidence of burnout. Participants who sometimes used WeChat Moments had the lowest incidence of overall burnout and DP, and those who usually used WeChat Moments had the lowest incidence of EE.

**Table 2.** Association between WeChat Moments usage and burnout (N=3769).

WeChat Moments usage	Overall burnout^a^		Emotional exhaustion^b^		Depersonalization^c^		Low personal accomplishment^d^	
	Yes	No	Yes	No	Yes	No	Yes	No
Never, n (%)	35 (28.0)	90 (72.0)	53 (42.4)	72 (57.6)	49 (39.2)	76 (60.8)	85 (68.0)	40 (32.0)
Seldom, n (%)	103 (19.9)	415 (80.1)	179 (34.6)	339 (65.4)	170 (32.8)	348 (67.2)	337 (65.1)	181 (34.9)
Occasionally, n (%)	315 (16.8)	1556 (83.2)	561 (30.0)	1310 (70.0)	539 (28.8)	1332 (71.2)	1209 (64.6)	662 (35.4)
Sometimes, n (%)	117 (13.3)	762 (86.7)	231 (26.3)	648 (73.7)	204 (23.2)	675 (76.8)	519 (59.0)	360 (41.0)
Usually, n (%)	55 (14.6)	321 (85.4)	96 (25.5)	280 (74.5)	95 (25.3)	281 (74.7)	221 (58.8)	155 (41.2)

a *χ*2_4_=23.790; *P*<.001.

b *χ*2_4_=23.624; *P*<.001.

c *χ*2_4_=25.727; *P*<.001.

d *χ*2_4_=13.133; *P*=.01.

### Multivariate Logistic Regression Results of the Effect of WeChat Moments Usage on Burnout

In model 1, we adjusted for sociodemographic factors, and the subsequent models were gradually adjusted for individual health factors, occupational status factors, and COVID-19 pandemic factors. The multivariate logistic regression results of the effect of WeChat Moments usage on burnout are presented in Table 3. Regarding overall burnout, compared with people who never used WeChat Moments, those who used WeChat Moments had a lower probability of overall burnout in models 1-3, while only those who sometimes used WeChat Moments had a lower probability of overall burnout with adjustment for COVID-19 pandemic factors. Regarding EE, those who used WeChat Moments had a lower probability of EE in models 1-3, and there was no significant (all *P*>.05) finding for the frequency of using WeChat Moments and the occurrence of EE in model 4. Regarding DP, participants who occasionally, sometimes, or usually used WeChat Moments had a lower probability of DP in models 1 and 2. Moreover, participants who sometimes or usually used WeChat Moments had a lower probability of DP in model 3, and there was no significant finding for the frequency of using WeChat Moments and the occurrence of DP in model 4 (all *P*>.05). Regarding PA, only in model 1, participants who sometimes or usually used WeChat Moments had a lower probability of experiencing low PA. The specific data of models 1‐3 are shown in Multimedia Appendix 2.

**Table 3.** Multivariate logistic regression results of the effect of WeChat Moments usage on burnout.

Burnout category and WeChat Moments usage	Model 1^a^		Model 2^b^		Model 3^b^		Model 4^b^	
	OR^c^ (95% CI)	*P* value	OR (95% CI)	*P* value	OR (95% CI)	*P* value	OR (95% CI)	*P* value
Overall burnout								
Never	Ref^d^		Ref		Ref		Ref	
Seldom	0.576 (0.364-0.913)	.02	0.569 (0.355-0.911)	.02	0.580 (0.361-0.931)	.02	0.710 (0.432-1.170)	.18
Occasionally	0.490 (0.320-0.750)	.001	0.510 (0.330-0.789)	.002	0.525 (0.339-0.815)	.004	0.646 (0.406-1.027)	.06
Sometimes	0.385 (0.245-0.606)	<.001	0.424 (0.267-0.674)	<.001	0.436 (0.273-0.696)	.001	0.570 (0.348-0.933)	.03
Usually	0.413 (0.250-0.680)	.001	0.468 (0.280-0.782)	.004	0.485 (0.289-0.814)	.006	0.658 (0.381-1.134)	.13
Emotional exhaustion								
Never	Ref		Ref		Ref		Ref	
Seldom	0.642 (0.425-0.969)	.04	0.626 (0.408-0.960)	.03	0.621 (0.404-0.955)	.03	0.706 (0.446-1.117)	.14
Occasionally	0.552 (0.376-0.809)	.002	0.583 (0.391-0.868)	.008	0.583 (0.391-0.872)	.009	0.670 (0.436-1.029)	.07
Sometimes	0.472 (0.317-0.705)	<.001	0.544 (0.359-0.824)	.004	0.536 (0.353-0.815)	.004	0.669 (0.428-1.047)	.08
Usually	0.439 (0.283-0.680)	<.001	0.518 (0.328-0.818)	.005	0.518 (0.327-0.820)	.005	0.661 (0.405-1.080)	.10
Depersonalization								
Never	Ref		Ref		Ref		Ref	
Seldom	0.739 (0.487-1.123)	.16	0.738 (0.482-1.131)	.16	0.746 (0.486-1.145)	.18	0.875 (0.559-1.371)	.56
Occasionally	0.636 (0.431-0.938)	.02	0.669 (0.449-0.996)	.048	0.682 (0.457-1.017)	.06	0.802 (0.527-1.221)	.30
Sometimes	0.494 (0.328-0.742)	.001	0.539 (0.355-0.818)	.004	0.550 (0.362-0.837)	.005	0.683 (0.440-1.060)	.90
Usually	0.531 (0.340-0.827)	.005	0.597 (0.379-0.943)	.03	0.612 (0.387-0.967)	.04	0.784 (0.485-1.267)	.32
Personal accomplishment								
Never	Ref		Ref		Ref		Ref	
Seldom	0.828 (0.541-1.267)	.38	0.826 (0.537-1.269)	.38	0.828 (0.536-1.279)	.40	0.960 (0.610-1.511)	.86
Occasionally	0.795 (0.534-1.183)	.26	0.818 (0.547-1.224)	.33	0.819 (0.546-1.230)	.34	0.946 (0.618-1.446)	.80
Sometimes	0.651 (0.432-0.980)	.04	0.685 (0.452-1.037)	.07	0.694 (0.457-1.055)	.09	0.848 (0.548-1.314)	.46
Usually	0.609 (0.392-0.945)	.03	0.663 (0.424-1.035)	.07	0.668 (0.425-1.048)	.08	0.872 (0.544-1.397)	.57

a Model 1 controls for sociodemographic factors, including age, gender, marital status, education, living arrangements, and family relationships.

b Models 2, 3, and 4 respectively incorporate individual health factors (including smoking, drinking, disability, number of chronic diseases, and self-rated health), occupational status factors (including practice location, occupation, professional title, length of career in primary care, and monthly income), and COVID-19 pandemic factors (including frontline health workers, satisfaction with institutional provision of personal protective equipment, self-assessment of work intensity during COVID-19, and the Minnesota Satisfaction Questionnaire-Short Form).

c OR: odds ratio.

d Ref: reference.

### Relations Between WeChat Moments Usage and Burnout in Rural and Urban Areas

**Figure 1.**
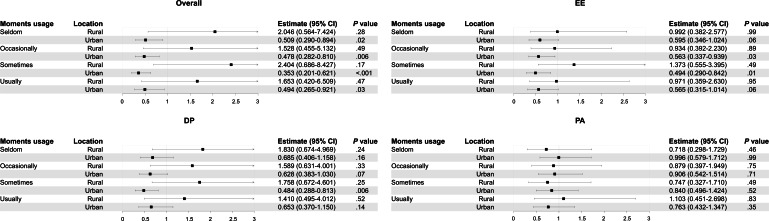
Relations between WeChat Moments usage and burnout among rural and urban areas. DP: depersonalization; EE: emotional exhaustion; PA: personal accomplishment.

We divided all participants into 2 groups by region, and Figure 1 shows the relations between WeChat Moments usage and burnout among the different regions with all factors adjusted. Compared with those who never used WeChat Moments, urban participants who used WeChat Moments had a lower probability of experiencing overall burnout, but rural participants had no significant findings (urban: all odds ratios and 95% CIs <1, and all *P*<.05; rural: all *P*>.05). Only urban PHWs who sometimes used WeChat Moments were less likely to experience EE. Moreover, only urban PHWs who sometimes used WeChat Moments were less likely to experience DP. There was no significant difference regarding PA in both rural and urban PHWs who used WeChat Moments compared with those who did not use WeChat Moments (urban: all *P*>.05; rural: all *P*>.05). The data obtained after subgrouping rural and urban areas in model 4 are shown in Multimedia Appendix 3.

## Discussion

### Principal Findings

Our study had 2 pivotal findings with public health implications. First, a pronounced urban-rural difference in burnout prevalence was observed among PHWs, with urban practitioners experiencing significantly higher prevalence. Second, moderated social media engagement, particularly through platforms like WeChat Moments, was associated with reduced burnout risks, though this relationship exhibited geographic heterogeneity.

In terms of overall burnout and various dimensions, the prevalence of PHWs was higher in urban areas than in rural areas, with significant urban-rural differences in the prevalence of overall burnout and EE. Another national study conducted in 2013 in China involving 4674 clinical physicians obtained similar results that the prevalence of burnout was higher in urban areas than in rural areas [33]. The differences found in this study may be attributed to the urban-rural dual structure in China. After decades of urbanization in China, the population in urban areas far exceeds that in rural areas. According to the data of the latest population statistics yearbook in China, there are 901,991,162 (63.89%) people in urban areas and 509,787,562 (36.11%) in rural areas [34]. However, the statistical bulletin of China’s health development in 2023 reported that the number of PHC personnel is higher in rural areas than in urban areas, with 3,823,000 general practitioners in total and 627,000 in urban areas [35], suggesting that the number of people under the care of each PHW is much higher in urban areas than in rural areas. Since our survey was conducted at the end of the COVID-19 pandemic, it was found that PHWs had undertaken a large amount of work throughout the pandemic for prevention and control, including maintaining the operations of PHC centers, building fever clinics, conducting epidemiological investigations, conducting vaccinations, and carrying out mass nucleic acid testing [36]. Due to the high population density and the fast transmission speed in cities, there were difficulties in pandemic prevention and high work pressures during the COVID-19 period. The research by Wang et al [33] showed that compared with rural clinicians, urban clinicians had a heavier workload and a higher incidence of burnout.

In our study of 3769 participants, a multivariate analysis adjusting for a range of covariates, encompassing sociodemographic, health-related, and occupational status factors, demonstrated that PHWs who used WeChat Moments were less likely to experience burnout than nonusers (seldom use versus never use: odds ratio 0.580, 95% CI 0.361‐0.931; *P*=.02). Specifically, the prevalence of EE and PA decreased as the frequency of WeChat Moments usage increased. Several independent studies support this finding. Fatima et al [37] found that ICU physicians’ use of social media relieved emotional stress and reduced burnout. Charoensukmongkol [38] demonstrated that social media use reduced burnout among employees with high levels of mindfulness. Moreover, a study of a closed Facebook group for female hematology or oncology physicians revealed that participation in this closed Facebook group reduced burnout compared with general Facebook participation [20]. The active participation of PHWs in social media platforms to alleviate burnout may stem from the multidimensional interactions that PHWs achieve through their WeChat Moments usage. PHWs, who are responsible for the health management of community residents or villagers, use WeChat Moments to share their weekly clinic hours, post health tips, and interact with and receive thumbs-ups from their fans, most of whom are community members living close to the community service center or village clinic. This positive feedback acknowledges their professional competence and enhances the relationship between health workers and community members, mitigating EE and augmenting social support and professional motivation [39]. However, it does not always mean that more frequent use of social media results in a lower prevalence of burnout. Two key findings in this study reveal the complex role of WeChat Moments usage.

First, the trend was not met regarding overall burnout and DP, and participants who sometimes used (less frequently) WeChat Moments benefited more than those who usually used (more frequently) WeChat Moments. This result is consistent with the finding of the Wuhan Citizen Study (n=320) in 2021 [40], which revealed that while social media provided informational and emotional succor, excessive health-related content exposure was linked to depression and secondary trauma (*P*<.01). Similar research evidence of a U-curve relationship between DP and social media use was obtained in samples of nonteaching employees at American and Thai universities, with the argument that moderate levels of social networking service use can help to reduce DP, and an intensity above this optimal level can increase DP [41]. This might be because social media has been described as a double-edged sword, offering benefits, such as informational, emotional, and peer support, but also posing significant risks due to overuse, which can lead to information overload and mental health problems [42,43]. In China’s PHW context, WeChat is often an obligatory tool for patient communication, health reporting, and administrative tasks. WeChat not only facilitates work and brings social connections, but also blurs the boundaries between work and life. The large number of work-related social connections on leisure social media may also erode professional identity, amplify emotional alienation, and further lead to DP. Thus, some studies have concluded that greater burnout is associated with greater use of social media [22,23]. Notably, our results also suggest that more than “sometimes” may indicate excessive and incorrect use of social media for overall burnout and DP. Moreover, studies have shown that there is a positive association between social media addiction and burnout [44-46]. It might be the tipping point between social media’s appropriate use and addictive use that causes the opposite role of social media in mental health. A cross-sectional study published in 2022 of 519 physicians in Hunan Province, China, showed that burnout had a significant positive effect on social media addiction (*β*=.300; *t*=6.621; 95% CI 0.191‐0.351; *P*<.01) [47]. A similar pattern was observed in a Turkish study of 529 physicians, where social media addiction levels increased as their burnout levels increased [48].

Second, the opposite results obtained in model 4 with the inclusion of COVID-19 and job satisfaction suggest that there was a potentially high-pressure environment during COVID-19 and that the influence of using social media in PHWs failed under the high pressure of the real environment (COVID-19) [49]. Moerdler et al [50] suggested that during the COVID-19 pandemic, it is unclear whether social media use contributed to burnout because burnout rates may have been related to potential or overall burnout.

Notably, our research showed an interesting result that the association between WeChat Moments usage and burnout was more significant in urban areas when participants were stratified by geographic location. On one hand, in a country like China that emphasizes family culture, family bonds and clan ties are more prominent in rural areas than in urban areas. According to the Seventh National Population Census, households with five or more members account for 8.12% of households in urban areas, 11.56% in towns, and 12.47% in rural areas. Meanwhile, multigenerational households (with grandparents, parents, and children co-residing) account for 11.31% of households in urban areas, 14.37% in towns, and 16.15% in rural areas. These statistics reveal two distinct patterns in China’s family structure: (1) larger household sizes persist in rural communities compared to urbanized areas, and (2) stronger intergenerational support systems are more prevalent in the countryside, as evidenced by the higher proportion of multigenerational cohabitation [51]. Family support is one of the most important factors in mitigating burnout [52], and rural residents usually have wider social networks and tend to seek solace from family support [53]. Therefore, the mitigating effect of WeChat Moments on burnout might not be significant at a time when rural PHWs can receive more family support. On the other hand, while online connections may substitute for face-to-face offline connections, such as family support, to some extent, they are not optimal in terms of providing well-being [54], with research suggesting that internet adoption improves personal well-being to a lesser extent than family ties [55]. This further suggests that PHWs living in rural areas with more extensive offline family support may be less likely to use social media to alleviate burnout, and this indicates that the content and intent behind WeChat Moments usage should be considered.

### Conclusion

Initially, our analysis disclosed an inverse association between the frequency of PHWs’ engagement with social media and the prevalence of burnout. However, this pattern was not mirrored in the overall burnout and DP dimensions, a result that underscores the distinct impacts of appropriate versus excessive social media use thresholds on burnout. Our findings indicate that the mitigating influence of social media is contingent upon geographic locale, exhibiting variations between urban and rural environments. In rural settings, the protective effect of social media was found to be insignificant.

### Implications

Our findings underscore the urgent need for policy makers and health care institutions to prioritize interventions addressing occupational burnout among PHWs, particularly in underresourced and high-stress settings. We have now entered the era of the internet, with the widespread adoption of smartphones heralding a new epoch. Our research indicates that the judicious use of social media could mitigate burnout among PHWs. In China’s context, where platforms like WeChat are deeply integrated into daily life and public health operations (eg, family doctor WeChat groups), health care institutions could develop moderated online communities where PHWs share achievements, receive public feedback, and access mental health resources. For example, local governments might pilot platforms that allow PHWs to showcase their work progress to the communities they serve, thereby strengthening trust and reducing EE. However, such initiatives must respect workload thresholds, as excessive social media demands could inadvertently exacerbate burnout. Moreover, interventions based on social media need to be context-specific, as their effectiveness may vary between urban and rural settings. In urban areas, strategies promoting the appropriate use of social media may benefit more, whereas rural areas might require alternative approaches, such as reinforcing family support systems. Further research is needed to explore the mechanisms by which social media can protect against burnout, particularly in diverse sociodemographic and geographic groups. As one of the first studies to explore the role of social media in PHW burnout within a middle-income country, our findings offer actionable insights for other low- and middle-income countries navigating similar challenges. International bodies (eg, the World Health Organization) should develop digital health guidelines specific to low- and middle-income countries to help policy makers balance the benefits and risks of social media.

### Limitations

A notable limitation of this study is its reliance on cross-sectional data, which allowed us to observe associations between WeChat Moments usage and burnout among PHWs during the COVID-19 pandemic, but did not enable us to establish causal relationships. The absence of longitudinal data precludes the examination of temporal dynamics and the determination of causality, including the potential for reverse causality and the bidirectional relationship between burnout and social media use. Moreover, the intensity of WeChat Moments usage, which is based on self-evaluation, lacks an objective basis for measurement. A more quantitative measure and a more detailed time frame, such as the number of hours spent on WeChat Moments per day or week, would provide a clearer indication of usage intensity. Lastly, the relationship between social media use and burnout in social comparisons should be further explored. Future research could benefit from longitudinal studies that track changes in social media use and burnout over time, as well as more objective measures of social media engagement.

## Supplementary material

10.2196/70398 Multimedia Appendix 1 Characteristics of the unweighted population (N=3769).

10.2196/70398 Multimedia Appendix 2 Multivariate logistic regression results of the effect of WeChat Moments usage on burnout in models 1-3.

10.2196/70398 Multimedia Appendix 3 Multivariate logistic regression results of the effect of WeChat Moments usage on burnout according to urban/rural division in model 4.

10.2196/70398 Checklist 1 STROBE (Strengthening the Reporting of Observational Studies in Epidemiology) checklist.

## Abbreviations

CHC: community health center
DP: depersonalization
EE: emotional exhaustion
MSQ-SF: Minnesota Satisfaction Questionnaire-Short Form
PA: personal accomplishment
PHC: primary health care
PHW: primary health care worker
STROBE: Strengthening the Reporting of Observational Studies in Epidemiology
