# Supplementary material for: Association Between Social Media Use and Burnout Among Primary Health Care Workers During the COVID-19 Pandemic in China: Nationwide Cross-Sectional Survey
Source: J Med Internet Res. 2025 Jul 31;27:e70398. doi: 10.2196/70398 (PMC12313347; doi:10.2196/70398)
Supplement: Multimedia Appendix 1 [file jmir-v27-e70398-s001.docx]

**Multimedia Appendix 1.** Characteristics of the unweighted population (N=3769).

| Characteristics | Total | Rural(n=989) | Urban(n=2780) | *x^2^*/*t* | *P* |
| --- | --- | --- | --- | --- | --- |
|  | n (%) | n (%) | n (%) |  |  |
| **Age (years)** |  |  |  |  |  |
| ≤30 | 974(25.8) | 278(28.1) | 696(25.0) | 12.372 | .006 |
| 31-40 | 1521(40.4) | 353(35.7) | 1168(42.0) |  |  |
| 41-50 | 880(23.3) | 244(24.7) | 636(22.9) |  |  |
| >50 | 394(10.5) | 114(11.5) | 280(10.1) |  |  |
| **Gender** |  |  |  |  |  |
| Male | 798(21.2) | 303(30.6) | 495(17.8) | 71.961 | <.001 |
| Female | 2971(78.8) | 686(69.4) | 2285(82.2) |  |  |
| **Marital status** |  |  |  |  |  |
| Single | 649(17.2) | 158(16.0) | 491(17.6) | 1.505 | .471 |
| Married | 2926(77.6) | 778(78.7) | 2148(77.3) |  |  |
| Divorced/Widowed | 194(5.2) | 53(5.3) | 141(5.1) |  |  |
| **Educational status** |  |  |  |  |  |
| High School or below | 407(10.8) | 226(22.9) | 181(6.5) | 210.688 | <.001 |
| Junior college | 1132(30.0) | 292(29.5) | 840(30.2) |  |  |
| Undergraduate or above | 2230(59.2) | 471(47.6) | 1759(63.3) |  |  |
| **Disability** |  |  |  |  |  |
| Yes | 89(2.4) | 25(2.5) | 64(2.3) | 0.161 | .688 |
| No | 3680(97.6) | 964(97.5) | 2716(97.7) |  |  |
| **Occupation category** |  |  |  |  |  |
| GP^a^ | 1174(31.1) | 418(42.2) | 756(27.2) | 88.605 | <.001 |
| Nurse | 1282(34.0) | 248(25.1) | 1034(37.2) |  |  |
| Public health physician | 253(6.7) | 62(6.3) | 191(6.9) |  |  |
| Managerial staff | 221(5.9) | 46(4.7) | 175(6.3) |  |  |
| Support staff | 839(22.3) | 215(21.7) | 624(22.4) |  |  |
| **MSQ-SF^b^ (Mean ± SD)** | 69.9±13.4 | 69.6±13.3 | 70.1±13.5 | 0.995 | .320 |
| **Moments usage** |  |  |  |  |  |
| Never | 125(3.3) | 38(3.9) | 87(3.1) | 14.542 | .006 |
| Seldom | 518(13.8) | 112(11.3) | 406(14.6) |  |  |
| Occasionally | 1871(49.6) | 522(52.8) | 1349(48.5) |  |  |
| Sometimes | 879(23.3) | 207(20.9) | 672(24.2) |  |  |
| Usually | 376(10.0) | 110(11.1) | 266(9.6) |  |  |
| **Burnout** |  |  |  |  |  |
| Overall | 625(16.6) | 135(13.7) | 490(17.6) | 8.336 | .004 |
| EE | 1120(29.7) | 245(24.8) | 875(31.5) | 15.690 | <.001 |
| DP | 1057(28.0) | 257(26.0) | 800(28.8) | 2.816c | .093 |
| PA | 2371(62.9) | 614(62.1) | 1757(63.2) | 0.391c | .532 |

^a^GP, general practitioner;

*^b^*MSQ-SF, Minnesota Satisfaction Questionnaire-Short Form.
