# Supplementary material for: Association Between Social Media Use and Burnout Among Primary Health Care Workers During the COVID-19 Pandemic in China: Nationwide Cross-Sectional Survey
Source: J Med Internet Res. 2025 Jul 31;27:e70398. doi: 10.2196/70398 (PMC12313347; doi:10.2196/70398)
Supplement: Multimedia Appendix 2 [file jmir-v27-e70398-s002.docx]

**Multimedia Appendix 2.** Multivariate logistic regression results of the effect of WeChat Moments usage on burnout in models 1-3.

**Table S1 Multivariate logistic regression result of effect of Moments usage on burnout in Table 3 Model 1**

|  | B | SE | Wald | v | P | OR | 95% CI | |
| --- | --- | --- | --- | --- | --- | --- | --- | --- |
|  |  |  |  |  |  |  |  | |
| Moments usage |  |  |  |  |  |  |  |  |
| Never |  |  | 20.693 | 4 | 0.000 |  |  |  |
| Seldom | -0.551 | 0.235 | 5.511 | 1 | 0.019 | 0.576 | 0.364 | 0.913 |
| Occasionally | -0.713 | 0.217 | 10.821 | 1 | 0.001 | 0.490 | 0.320 | 0.750 |
| Sometimes | -0.954 | 0.231 | 17.082 | 1 | 0.000 | 0.385 | 0.245 | 0.606 |
| Usually | -0.885 | 0.255 | 12.029 | 1 | 0.001 | 0.413 | 0.250 | 0.680 |
| Age (years) |  |  |  |  |  |  |  |  |
| ≤30 |  |  | 19.815 | 3 | 0.000 |  |  |  |
| 31-40 | -0.160 | 0.125 | 1.645 | 1 | 0.200 | 0.852 | 0.667 | 1.088 |
| 41-50 | -0.597 | 0.152 | 15.382 | 1 | 0.000 | 0.550 | 0.408 | 0.742 |
| >50 | -0.552 | 0.198 | 7.794 | 1 | 0.005 | 0.576 | 0.391 | 0.848 |
| Gender |  |  |  |  |  |  |  |  |
| Male |  |  |  |  |  |  |  |  |
| Female | -0.068 | 0.112 | 0.375 | 1 | 0.541 | 0.934 | 0.750 | 1.163 |
| Marital status |  |  |  |  |  |  |  |  |
| Single |  |  | 1.260 | 2 | 0.533 |  |  |  |
| Married | -0.048 | 0.146 | 0.105 | 1 | 0.746 | 0.954 | 0.716 | 1.271 |
| Divorced/Widowed | -0.286 | 0.260 | 1.211 | 1 | 0.271 | 0.751 | 0.451 | 1.251 |
| Educational status |  |  |  |  |  |  |  |  |
| High School or below |  |  | 6.782 | 2 | 0.034 |  |  |  |
| Junior college | 0.453 | 0.186 | 5.908 | 1 | 0.015 | 1.573 | 1.092 | 2.267 |
| Undergraduate or above | 0.460 | 0.180 | 6.538 | 1 | 0.011 | 1.584 | 1.113 | 2.252 |
| Living arrangement |  |  |  |  |  |  |  |  |
| Living alone |  |  | 2.080 | 2 | 0.353 |  |  |  |
| Living with family | -0.231 | 0.161 | 2.063 | 1 | 0.151 | 0.793 | 0.578 | 1.088 |
| Living with Others | -0.138 | 0.233 | .350 | 1 | 0.554 | 0.871 | 0.553 | 1.375 |
| Family relations |  |  |  |  |  |  |  |  |
| Poor |  |  | 19.965 | 2 | 0.000 |  |  |  |
| General | 0.221 | 0.245 | 0.814 | 1 | 0.367 | 1.247 | 0.772 | 2.015 |
| Good | -0.280 | 0.230 | 1.481 | 1 | 0.224 | 0.756 | 0.481 | 1.187 |

Note: Variables entered: Do you use WeChat to post on your Moments? , Age (4 categories), Gender, Marital status (3 categories), Educational status (3 categories), Living arrangement (3 categories), Family relations (3 categories).

**Table S2 Multivariate logistic regression result of effect of Moments usage on burnout in Table 3 Model 2**

|  | B | SE | Wald | v | p | Exp(B) | 95% CI | |
| --- | --- | --- | --- | --- | --- | --- | --- | --- |
|  |  |  |  |  |  |  |  | |
| Moments usage |  |  |  |  |  |  |  |  |
| Never |  |  | 14.237 | 4 | 0.007 |  |  |  |
| Seldom | -0.564 | 0.240 | 5.524 | 1 | 0.019 | 0.569 | 0.355 | 0.911 |
| Occasionally | -0.673 | 0.222 | 9.144 | 1 | 0.002 | 0.510 | 0.330 | 0.789 |
| Sometimes | -0.858 | 0.237 | 13.132 | 1 | 0.000 | 0.424 | 0.267 | 0.674 |
| Usually | -0.760 | 0.262 | 8.400 | 1 | 0.004 | 0.468 | 0.280 | 0.782 |
| Sociodemographic |  |  |  |  |  |  |  |  |
| Age (years) |  |  |  |  |  |  |  |  |
| ≤30 |  |  | 17.706 | 3 | 0.001 |  |  |  |
| 31-40 | -0.191 | 0.128 | 2.230 | 1 | 0.135 | 0.827 | 0.644 | 1.061 |
| 41-50 | -0.616 | 0.158 | 15.304 | 1 | 0.000 | 0.540 | 0.396 | 0.735 |
| >50 | -0.525 | 0.208 | 6.377 | 1 | 0.012 | 0.591 | 0.393 | 0.889 |
| Gender |  |  |  |  |  |  |  |  |
| Male |  |  |  |  |  |  |  |  |
| Female | -0.219 | 0.136 | 2.610 | 1 | 0.106 | 0.803 | 0.616 | 1.048 |
| Marital status |  |  |  |  |  |  |  |  |
| Single |  |  | 1.222 | 2 | 0.543 |  |  |  |
| Married | -0.051 | 0.150 | 0.118 | 1 | 0.731 | 0.950 | 0.709 | 1.274 |
| Divorced/Widowed | -0.287 | 0.264 | 1.182 | 1 | 0.277 | 0.751 | 0.448 | 1.259 |
| Educational status |  |  |  |  |  |  |  |  |
| High School or below |  |  | 4.280 | 2 | 0.118 |  |  |  |
| Junior college | 0.391 | 0.189 | 4.272 | 1 | 0.039 | 1.479 | 1.020 | 2.143 |
| Undergraduate or above | 0.330 | 0.183 | 3.242 | 1 | 0.072 | 1.391 | 0.971 | 1.992 |
| Living arrangement |  |  |  |  |  |  |  |  |
| Living alone |  |  | 2.546 | 2 | 0.280 |  |  |  |
| Living with family | -0.262 | 0.164 | 2.544 | 1 | 0.111 | 0.769 | 0.557 | 1.062 |
| Living with Others | -0.176 | 0.237 | 0.552 | 1 | 0.457 | 0.839 | 0.528 | 1.334 |
| Family relations |  |  |  |  |  |  |  |  |
| Poor |  |  | 9.529 | 2 | 0.009 |  |  |  |
| General | 0.179 | 0.252 | 0.503 | 1 | 0.478 | 1.196 | 0.729 | 1.962 |
| Good | -0.177 | 0.239 | 0.551 | 1 | 0.458 | 0.838 | 0.525 | 1.337 |
| Individual health |  |  |  |  |  |  |  |  |
| Smoking |  |  |  |  |  |  |  |  |
| Non-smoker |  |  | 6.115 | 2 | 0.047 |  |  |  |
| Once, now quit | 0.217 | 0.275 | 0.622 | 1 | 0.430 | 1.242 | 0.725 | 2.127 |
| Current smoking | -0.550 | 0.257 | 4.595 | 1 | 0.032 | 0.577 | 0.349 | 0.954 |
| Drinking |  |  |  |  |  |  |  |  |
| Non-drinker |  |  | 0.900 | 2 | 0.638 |  |  |  |
| Once, now quit | 0.170 | 0.214 | 0.634 | 1 | 0.426 | 1.186 | 0.779 | 1.804 |
| Current drinking | 0.115 | 0.176 | 0.432 | 1 | 0.511 | 1.122 | 0.796 | 1.583 |
| Disability |  |  |  |  |  |  |  |  |
| Yes |  |  |  |  |  |  |  |  |
| No | 0.249 | 0.271 | 0.840 | 1 | 0.359 | 1.282 | 0.754 | 2.182 |
| Number of chronic diseases |  |  |  |  |  |  |  |  |
| 0 |  |  | 3.123 | 2 | 0.210 |  |  |  |
| 1 | -0.233 | 0.138 | 2.842 | 1 | 0.092 | 0.792 | 0.604 | 1.039 |
| 2 and above | -0.179 | 0.198 | 0.822 | 1 | 0.365 | 0.836 | 0.567 | 1.232 |
| Self-rated health |  |  |  |  |  |  |  |  |
| Bad |  |  | 68.399 | 2 | 0.000 |  |  |  |
| General | -0.458 | 0.136 | 11.275 | 1 | 0.001 | 0.633 | 0.484 | 0.826 |
| Good | -1.248 | 0.163 | 58.358 | 1 | 0.000 | 0.287 | 0.208 | 0.395 |

Note: Variables entered: Do you use WeChat to post on your Moments? , Age (4 categories), Gender, Marital status (3 categories), Educational status (3 categories), Living arrangement (3 categories), Family relations (3 categories), Do you smoke, Do you drink alcohol, Do you have a physical disability? , Number of chronic diseases you are suffering from (chronic diseases are: hypertension, hyperlipidemia, coronary heart disease, stroke, diabetes, malignant tumor, chronic obstructive pulmonary disease, chronic kidney disease, arthritis), Self-rated health (3 categories).

**Table S3 Multivariate logistic regression result of effect of Moments usage on burnout in Table 3 Model 3**

|  | B | SE | Wald | v | p | Exp(B) | 95% CI | |
| --- | --- | --- | --- | --- | --- | --- | --- | --- |
| Moments usage |  |  |  |  |  |  |  |  |
| Never |  |  | 13.048 | 4 | 0.011 |  |  |  |
| Seldom | -0.545 | 0.241 | 5.093 | 1 | 0.024 | 0.580 | 0.361 | 0.931 |
| Occasionally | -0.644 | 0.224 | 8.257 | 1 | 0.004 | 0.525 | 0.339 | 0.815 |
| Sometimes | -0.829 | 0.238 | 12.094 | 1 | 0.001 | 0.436 | 0.273 | 0.696 |
| Usually | -0.723 | 0.264 | 7.515 | 1 | 0.006 | 0.485 | 0.289 | 0.814 |
| Sociodemographic |  |  |  |  |  |  |  |  |
| Age (years) |  |  | 4.678 | 3 | 0.197 |  |  |  |
| ≤30 | -0.130 | 0.140 | 0.861 | 1 | 0.354 | 0.878 | 0.667 | 1.156 |
| 31-40 | -0.448 | 0.213 | 4.430 | 1 | 0.035 | 0.639 | 0.421 | 0.970 |
| 41-50 | -0.334 | 0.277 | 1.451 | 1 | 0.228 | 0.716 | 0.416 | 1.233 |
| >50 |  |  |  |  |  |  |  |  |
| Gender |  |  |  |  |  |  |  |  |
| Male |  |  |  |  |  |  |  |  |
| Female | -0.328 | 0.145 | 5.131 | 1 | 0.024 | 0.720 | 0.542 | 0.957 |
| Marital status |  |  |  |  |  |  |  |  |
| Single |  |  | 1.212 | 2 | 0.546 |  |  |  |
| Married | -0.033 | 0.151 | 0.046 | 1 | 0.830 | 0.968 | 0.719 | 1.302 |
| Divorced/Widowed | -0.279 | 0.265 | 1.104 | 1 | 0.293 | 0.757 | 0.450 | 1.273 |
| Educational status |  |  |  |  |  |  |  |  |
| High School or below |  |  | 3.142 | 2 | 0.208 |  |  |  |
| Junior college | 0.343 | 0.197 | 3.049 | 1 | 0.081 | 1.409 | 0.959 | 2.072 |
| Undergraduate or above | 0.332 | 0.204 | 2.656 | 1 | 0.103 | 1.393 | 0.935 | 2.077 |
| Living arrangement |  |  |  |  |  |  |  |  |
| Living alone |  |  | 2.723 | 2 | 0.256 |  |  |  |
| Living with family | -0.273 | 0.166 | 2.721 | 1 | 0.099 | 0.761 | 0.550 | 1.053 |
| Living with Others | -0.200 | 0.238 | 0.710 | 1 | 0.399 | 0.818 | 0.513 | 1.304 |
| Family relations |  |  |  |  |  |  |  |  |
| Poor |  |  | 9.464 | 2 | 0.009 |  |  |  |
| General | 0.211 | 0.254 | 0.692 | 1 | 0.406 | 1.235 | 0.751 | 2.032 |
| Good | -0.148 | 0.240 | 0.379 | 1 | 0.538 | 0.862 | 0.538 | 1.382 |
| Individual health |  |  |  |  |  |  |  |  |
| Smoking |  |  |  |  |  |  |  |  |
| Non-smoker |  |  | 6.589 | 2 | 0.037 |  |  |  |
| Once, now quit | 0.253 | 0.276 | 0.837 | 1 | 0.360 | 1.288 | 0.749 | 2.213 |
| Current smoking | -0.562 | 0.259 | 4.716 | 1 | 0.030 | 0.570 | 0.343 | 0.947 |
| Drinking |  |  |  |  |  |  |  |  |
| Non-drinker |  |  | 1.022 | 2 | 0.600 |  |  |  |
| Once, now quit | 0.193 | 0.216 | 0.802 | 1 | 0.370 | 1.213 | 0.795 | 1.853 |
| Current drinking | 0.111 | 0.176 | 0.398 | 1 | 0.528 | 1.118 | 0.791 | 1.579 |
| Disability |  |  |  |  |  |  |  |  |
| Yes |  |  |  |  |  |  |  |  |
| No | 0.298 | 0.273 | 1.193 | 1 | 0.275 | 1.348 | 0.789 | 2.301 |
| Number of chronic diseases |  |  |  |  |  |  |  |  |
| 0 |  |  | 3.043 | 2 | 0.218 |  |  |  |
| 1 | -0.229 | 0.139 | 2.713 | 1 | 0.100 | 0.795 | 0.605 | 1.045 |
| 2 and above | -0.189 | 0.199 | 0.898 | 1 | 0.343 | 0.828 | 0.560 | 1.224 |
| Self-rated health |  |  |  |  |  |  |  |  |
| Bad |  |  | 65.230 | 2 | 0.000 |  |  |  |
| General | -0.455 | 0.137 | 11.025 | 1 | 0.001 | 0.634 | 0.485 | 0.830 |
| Good | -1.230 | 0.165 | 55.845 | 1 | 0.000 | 0.292 | 0.212 | 0.404 |
| Practice location |  |  |  |  |  |  |  |  |
| Rural area |  |  |  |  |  |  |  |  |
| Urban area | 0.173 | 0.115 | 2.275 | 1 | 0.132 | 1.189 | 0.949 | 1.489 |
| Occupation category |  |  |  |  |  |  |  |  |
| GP |  |  | 5.576 | 4 | .233 |  |  |  |
| Nurse | 0.220 | 0.129 | 2.885 | 1 | 0.089 | 1.246 | 0.967 | 1.605 |
| Public health physician | -0.052 | 0.204 | 0.066 | 1 | 0.798 | 0.949 | 0.636 | 1.416 |
| Managerial staff | -0.032 | 0.226 | 0.020 | 1 | 0.887 | 0.968 | 0.622 | 1.508 |
| Support staff | 0.236 | 0.132 | 3.199 | 1 | 0.074 | 1.266 | 0.978 | 1.639 |
| Professional title |  |  |  |  |  |  |  |  |
| Not rated |  |  | 0.626 | 3 | 0.890 |  |  |  |
| Junior | 0.026 | 0.146 | 0.032 | 1 | 0.858 | 1.026 | 0.770 | 1.368 |
| Intermediate grade | 0.087 | 0.175 | 0.245 | 1 | 0.621 | 1.091 | 0.773 | 1.538 |
| Senior | -0.048 | 0.266 | 0.032 | 1 | 0.858 | 0.953 | 0.566 | 1.607 |
| Length of career in primary care (years) |  |  |  |  |  |  |  |  |
| ≤10 |  |  | 1.769 | 2 | 0.413 |  |  |  |
| 11-20 | -0.168 | 0.129 | 1.701 | 1 | 0.192 | 0.845 | 0.657 | 1.088 |
| > 20 | -0.166 | 0.206 | 0.647 | 1 | 0.421 | 0.847 | 0.566 | 1.269 |
| Monthly income (CNY) |  |  |  |  |  |  |  |  |
| <3000 |  |  | 0.425 | 2 | 0.809 |  |  |  |
| 3000-5000 | -0.078 | 0.120 | 0.420 | 1 | 0.517 | 0.925 | 0.730 | 1.171 |
| >5000 | -0.067 | 0.143 | 0.219 | 1 | 0.640 | 0.935 | 0.707 | 1.238 |

Note: Variables entered: Do you use WeChat to post on your Moments? , Age (4 categories), Gender, Marital status (3 categories), Educational status (3 categories), Living arrangement (3 categories), Family relations (3 categories), Do you smoke, Do you drink alcohol, Do you have a physical disability? , Number of chronic diseases you are suffering from (chronic diseases are: hypertension, hyperlipidemia, coronary heart disease, stroke, diabetes, malignant tumor, chronic obstructive pulmonary disease, chronic kidney disease, arthritis), Self-rated health (3 categories), Practice location, Occupation category (5 categories), Professional title (4 categories), Length of career in primary care (3 categories), Monthly income (3 categories).
