# Supplementary material for: Association Between Social Media Use and Burnout Among Primary Health Care Workers During the COVID-19 Pandemic in China: Nationwide Cross-Sectional Survey
Source: J Med Internet Res. 2025 Jul 31;27:e70398. doi: 10.2196/70398 (PMC12313347; doi:10.2196/70398)
Supplement: Multimedia Appendix 3 [file jmir-v27-e70398-s003.docx]

**Multimedia Appendix 3.** Multivariate logistic regression results of the effect of WeChat Moments usage on burnout according to urban/rural division in model 4.

**Table S1 Multivariate logistic regression result of effect of Moments usage on burnout in rural areas**

|  | B | SE | Wald | v | p | Exp(B) | 95% CI | |
| --- | --- | --- | --- | --- | --- | --- | --- | --- |
|  |  |  |  |  |  |  |  | |
| Moments usage |  |  | 4.268 | 4 | .371 |  |  |  |
| Never |  |  | 13.048 | 4 | 0.011 |  |  |  |
| Seldom | .716 | .658 | 1.185 | 1 | .276 | 2.046 | .564 | 7.424 |
| Occasionally | .424 | .618 | .470 | 1 | .493 | 1.528 | .455 | 5.132 |
| Sometimes | .877 | .640 | 1.878 | 1 | .171 | 2.404 | .686 | 8.427 |
| Usually | .503 | .699 | .517 | 1 | .472 | 1.653 | .420 | 6.509 |
| Sociodemographic |  |  |  |  |  |  |  |  |
| Age (years) |  |  |  |  |  |  |  |  |
| ≤30 |  |  | 3.465 | 3 | .325 |  |  |  |
| 31-40 | -.351 | .314 | 1.251 | 1 | .263 | .704 | .381 | 1.302 |
| 41-50 | -.764 | .535 | 2.038 | 1 | .153 | .466 | .163 | 1.329 |
| >50 | -.189 | .659 | .082 | 1 | .774 | .828 | .227 | 3.013 |
| Gender |  |  |  |  |  |  |  |  |
| Male |  |  |  |  |  |  |  |  |
| Female | -.298 | .313 | .905 | 1 | .342 | .742 | .402 | 1.372 |
| Marital status |  |  |  |  |  |  |  |  |
| Single |  |  | 8.532 | 2 | .014 |  |  |  |
| Married | -.862 | .303 | 8.084 | 1 | .004 | .422 | .233 | .765 |
| Divorced/Widowed | -1.129 | .676 | 2.792 | 1 | .095 | .323 | .086 | 1.216 |
| Educational status |  |  |  |  |  |  |  |  |
| High School or below |  |  | 1.211 | 2 | .546 |  |  |  |
| Junior college | .309 | .343 | .812 | 1 | .367 | 1.362 | .696 | 2.667 |
| Undergraduate or above | .080 | .370 | .047 | 1 | .828 | 1.084 | .524 | 2.240 |
| Living arrangement |  |  |  |  |  |  |  |  |
| Living alone |  |  | .008 | 2 | .996 |  |  |  |
| Living with family | -.012 | .322 | .001 | 1 | .969 | .988 | .525 | 1.857 |
| Living with Others | .026 | .522 | .002 | 1 | .961 | 1.026 | .369 | 2.852 |
| Family relations |  |  |  |  |  |  |  |  |
| Poor |  |  | 10.109 | 2 | .006 |  |  |  |
| General | .673 | .708 | .902 | 1 | .342 | 1.960 | .489 | 7.856 |
| Good | -.144 | .700 | .042 | 1 | .837 | .866 | .219 | 3.415 |
| Individual health |  |  |  |  |  |  |  |  |
| Smoking |  |  |  |  |  |  |  |  |
| Non-smoker |  |  | 3.445 | 2 | .179 |  |  |  |
| Once, now quit | .070 | .565 | .015 | 1 | .902 | 1.072 | .354 | 3.244 |
| Current smoking | -.945 | .526 | 3.231 | 1 | .072 | .389 | .139 | 1.089 |
| Drinking |  |  |  |  |  |  |  |  |
| Non-drinker |  |  | .132 | 2 | .936 |  |  |  |
| Once, now quit | -.052 | .409 | .016 | 1 | .899 | .949 | .425 | 2.118 |
| Current drinking | -.139 | .387 | .129 | 1 | .720 | .870 | .407 | 1.860 |
| Disability |  |  |  |  |  |  |  |  |
| Yes |  |  |  |  |  |  |  |  |
| No | .445 | .611 | .532 | 1 | .466 | 1.561 | .472 | 5.166 |
| Number of chronic diseases |  |  |  |  |  |  |  |  |
| 0 |  |  | 1.683 | 2 | .431 |  |  |  |
| 1 | -.313 | .354 | .781 | 1 | .377 | .732 | .366 | 1.463 |
| 2 and above | .351 | .463 | .574 | 1 | .449 | 1.420 | .573 | 3.522 |
| Self-rated health |  |  |  |  |  |  |  |  |
| Bad |  |  | 1.490 | 2 | .475 |  |  |  |
| General | -.086 | .383 | .051 | 1 | .821 | .917 | .433 | 1.942 |
| Good | -.354 | .420 | .710 | 1 | .399 | .702 | .308 | 1.599 |
| Occupation category |  |  |  |  |  |  |  |  |
| GP |  |  | 5.757 | 4 | .218 |  |  |  |
| Nurse | .039 | .296 | .018 | 1 | .895 | 1.040 | .582 | 1.859 |
| Public health physician | .424 | .432 | .960 | 1 | .327 | 1.528 | .655 | 3.565 |
| Managerial staff | -.069 | .596 | .014 | 1 | .907 | .933 | .290 | 2.998 |
| Support staff | .559 | .272 | 4.229 | 1 | .040 | 1.749 | 1.027 | 2.980 |
| Professional title |  |  |  |  |  |  |  |  |
| Not rated |  |  | 2.796 | 3 | .424 |  |  |  |
| Junior | .086 | .280 | .094 | 1 | .759 | 1.090 | .630 | 1.885 |
| Intermediate grade | .468 | .350 | 1.792 | 1 | .181 | 1.598 | .805 | 3.172 |
| Senior | .643 | .541 | 1.415 | 1 | .234 | 1.903 | .659 | 5.494 |
| Length of career in primary care (years) |  |  |  |  |  |  |  |  |
| ≤10 |  |  | .917 | 2 | .632 |  |  |  |
| 11-20 | -.266 | .326 | .667 | 1 | .414 | .766 | .404 | 1.452 |
| > 20 | -.432 | .533 | .657 | 1 | .418 | .649 | .229 | 1.844 |
| Monthly income (CNY) |  |  |  |  |  |  |  |  |
| <3000 |  |  | .167 | 2 | .920 |  |  |  |
| 3000-5000 | .099 | .258 | .149 | 1 | .700 | 1.105 | .666 | 1.831 |
| >5000 | .026 | .331 | .006 | 1 | .938 | 1.026 | .536 | 1.964 |
| COVID-19 pandemic |  |  |  |  |  |  |  |  |
| Front-line health workers |  |  |  |  |  |  |  |  |
| Yes |  |  |  |  |  |  |  |  |
| No | -.321 | .491 | .426 | 1 | .514 | .726 | .277 | 1.901 |
| Satisfaction with institutional provision of PPE |  |  |  |  |  |  |  |  |
| Low |  |  | 4.047 | 2 | .132 |  |  |  |
| Medium | .884 | .547 | 2.612 | 1 | .106 | 2.420 | .829 | 7.071 |
| High | .436 | .519 | .704 | 1 | .401 | 1.546 | .559 | 4.279 |
| Self-rated work intensity during COVID-19 |  |  |  |  |  |  |  |  |
| Low |  |  | .400 | 2 | .819 |  |  |  |
| Medium | .476 | 1.144 | .173 | 1 | .677 | 1.610 | .171 | 15.145 |
| High | .574 | 1.146 | .251 | 1 | .617 | 1.775 | .188 | 16.777 |
| MSQ-SF (Mean ± SD) | -.032 | .009 | 12.377 | 1 | .000 | .969 | .952 | .986 |

**Table S2 Multivariate logistic regression result of effect of Moments usage on burnout in urban areas**

|  | B | SE | Wald | v | p | Exp(B) | 95% CI | |
| --- | --- | --- | --- | --- | --- | --- | --- | --- |
| Moments usage |  |  | 4.268 | 4 | .371 |  |  |  |
| Never |  |  | 14.082 | 4 | .007 |  |  |  |
| Seldom | -.675 | .287 | 5.525 | 1 | .019 | .509 | .290 | .894 |
| Occasionally | -.737 | .269 | 7.530 | 1 | .006 | .478 | .282 | .810 |
| Sometimes | -1.040 | .287 | 13.095 | 1 | .000 | .353 | .201 | .621 |
| Usually | -.706 | .318 | 4.929 | 1 | .026 | .494 | .265 | .921 |
| Sociodemographic |  |  |  |  |  |  |  |  |
| Age (years) |  |  |  |  |  |  |  |  |
| ≤30 |  |  | 2.859 | 3 | .414 |  |  |  |
| 31-40 | -.137 | .169 | .652 | 1 | .419 | .872 | .626 | 1.216 |
| 41-50 | -.401 | .250 | 2.565 | 1 | .109 | .670 | .410 | 1.094 |
| >50 | -.239 | .326 | .535 | 1 | .464 | .788 | .416 | 1.493 |
| Gender |  |  |  |  |  |  |  |  |
| Male |  |  |  |  |  |  |  |  |
| Female | -.303 | .176 | 2.961 | 1 | .085 | .738 | .523 | 1.043 |
| Marital status |  |  |  |  |  |  |  |  |
| Single |  |  | 1.903 | 2 | .386 |  |  |  |
| Married | .191 | .191 | 1.003 | 1 | .317 | 1.211 | .833 | 1.760 |
| Divorced/Widowed | -.096 | .313 | .093 | 1 | .760 | .909 | .492 | 1.680 |
| Educational status |  |  |  |  |  |  |  |  |
| High School or below |  |  | 1.016 | 2 | .602 |  |  |  |
| Junior college | .236 | .265 | .792 | 1 | .374 | 1.266 | .753 | 2.130 |
| Undergraduate or above | .148 | .272 | .295 | 1 | .587 | 1.159 | .680 | 1.975 |
| Living arrangement |  |  |  |  |  |  |  |  |
| Living alone |  |  | 3.837 | 2 | .147 |  |  |  |
| Living with family | -.379 | .213 | 3.173 | 1 | .075 | .685 | .451 | 1.039 |
| Living with Others | -.051 | .288 | .032 | 1 | .858 | .950 | .540 | 1.672 |
| Family relations |  |  |  |  |  |  |  |  |
| Poor |  |  | 1.188 | 2 | .552 |  |  |  |
| General | .310 | .311 | .995 | 1 | .319 | 1.363 | .741 | 2.507 |
| Good | .320 | .294 | 1.183 | 1 | .277 | 1.377 | .774 | 2.451 |
| Individual health |  |  |  |  |  |  |  |  |
| Smoking |  |  |  |  |  |  |  |  |
| Non-smoker |  |  | 4.925 | 2 | .085 |  |  |  |
| Once, now quit | .366 | .360 | 1.035 | 1 | .309 | 1.442 | .712 | 2.921 |
| Current smoking | -.578 | .335 | 2.988 | 1 | .084 | .561 | .291 | 1.080 |
| Drinking |  |  |  |  |  |  |  |  |
| Non-drinker |  |  | .510 | 2 | .775 |  |  |  |
| Once, now quit | .166 | .276 | .363 | 1 | .547 | 1.181 | .688 | 2.028 |
| Current drinking | .105 | .217 | .236 | 1 | .627 | 1.111 | .726 | 1.701 |
| Disability |  |  |  |  |  |  |  |  |
| Yes |  |  |  |  |  |  |  |  |
| No | .107 | .337 | .100 | 1 | .751 | 1.113 | .575 | 2.155 |
| Number of chronic diseases |  |  |  |  |  |  |  |  |
| 0 |  |  | 3.661 | 2 | .160 |  |  |  |
| 1 | -.270 | .161 | 2.812 | 1 | .094 | .763 | .557 | 1.047 |
| 2 and above | -.305 | .236 | 1.667 | 1 | .197 | .737 | .464 | 1.171 |
| Self-rated health |  |  |  |  |  |  |  |  |
| Bad |  |  | 17.286 | 2 | .000 |  |  |  |
| General | -.037 | .162 | .052 | 1 | .820 | .964 | .702 | 1.324 |
| Good | -.631 | .204 | 9.588 | 1 | .002 | .532 | .357 | .793 |
| Occupation category |  |  |  |  |  |  |  |  |
| GP |  |  | 4.718 | 4 | .317 |  |  |  |
| Nurse | .213 | .155 | 1.882 | 1 | .170 | 1.237 | .913 | 1.677 |
| Public health physician | -.254 | .248 | 1.050 | 1 | .305 | .776 | .477 | 1.261 |
| Managerial staff | -.056 | .262 | .046 | 1 | .830 | .945 | .566 | 1.579 |
| Support staff | .088 | .165 | .287 | 1 | .592 | 1.092 | .791 | 1.508 |
| Professional title |  |  |  |  |  |  |  |  |
| Not rated |  |  | 1.832 | 3 | .608 |  |  |  |
| Junior | -.162 | .190 | .723 | 1 | .395 | .851 | .586 | 1.235 |
| Intermediate grade | -.258 | .221 | 1.357 | 1 | .244 | .773 | .501 | 1.192 |
| Senior | -.410 | .327 | 1.574 | 1 | .210 | .664 | .350 | 1.259 |
| Length of career in primary care (years) |  |  |  |  |  |  |  |  |
| ≤10 |  |  | .839 | 2 | .657 |  |  |  |
| 11-20 | -.137 | .151 | .819 | 1 | .366 | .872 | .649 | 1.173 |
| > 20 | -.126 | .237 | .283 | 1 | .595 | .881 | .554 | 1.403 |
| Monthly income (CNY) |  |  |  |  |  |  |  |  |
| <3000 |  |  | 1.125 | 2 | .570 |  |  |  |
| 3000-5000 | -.003 | .149 | .001 | 1 | .982 | .997 | .744 | 1.335 |
| >5000 | .134 | .173 | .599 | 1 | .439 | 1.144 | .814 | 1.606 |
| COVID-19 pandemic |  |  |  |  |  |  |  |  |
| Front-line health workers |  |  |  |  |  |  |  |  |
| Yes |  |  |  |  |  |  |  |  |
| No | .188 | .348 | .292 | 1 | .589 | 1.207 | .610 | 2.386 |
| Satisfaction with institutional provision of PPE |  |  |  |  |  |  |  |  |
| Low |  |  | 19.522 | 2 | .000 |  |  |  |
| Medium | -.378 | .281 | 1.811 | 1 | .178 | .686 | .396 | 1.188 |
| High | -.848 | .269 | 9.915 | 1 | .002 | .428 | .253 | .726 |
| Self-rated work intensity during COVID-19 |  |  |  |  |  |  |  |  |
| Low |  |  | 9.274 | 2 | .010 |  |  |  |
| Medium | -.242 | .972 | .062 | 1 | .803 | .785 | .117 | 5.272 |
| High | .175 | .969 | .032 | 1 | .857 | 1.191 | .178 | 7.955 |
| MSQ-SF (Mean ± SD) | -.051 | .005 | 97.643 | 1 | .000 | .950 | .940 | .960 |
